# Emerging Threat of *Wickerhamomyces anomalous* (Formerly *Candida pelliculosa*) in Neonates: A Case Series and Review of Current Evidence

**DOI:** 10.1155/crcc/3715878

**Published:** 2026-08-17

**Authors:** Dipika Shaw, Gargi Mudey, Supriya Meshram, Deependra Manish, Aditi Warghade, Dhruba Hari Chandi

**Affiliations:** ^1^ Department of Microbiology, Jawaharlal Nehru Medical College, Datta Meghe Institute of Higher Education and Research, Wardha, India, dmimsu.edu.in

**Keywords:** AFST, antifungals, neonatal sepsis, VITEK 2 system, *Wickerhamomyces anomalous*

## Abstract

**Introduction:**

Neonatal sepsis is a systemic infection in newborn infants within the first month of life. It is a serious condition with a higher rate of mortality, thus it requires prompt recognition and treatment. Invasive candidiasis (IC) is a substantial source of morbidity and mortality in the neonatal intensive care unit (NICU) and the third most frequent cause of late‐onset sepsis in very‐low‐birth‐weight (VLBW) newborns. For the IC, the major etiological agent is *Candida albicans,* but other nonalbicans *Candida* (NAC) species can also invade the bloodstream and spread the infection. In a recent scenario, the outbreak of *Wickerhamomyces anomalous* (formerly *C. pelliculosa*) fungemia in a NICU presents a significant challenge in healthcare settings. This case report series presents six cases of *W. anomalous* sepsis in neonates admitted to our NICU.

**Case report:**

The study investigated the clinical and treatment profile of six cases of fungemia originating from the NICU. The objective was to assess the therapy outcome. An automated system (VITEK 2) was used for rapid biochemical identification of *Candida* species, which revealed *W. anomalous* growth. Furthermore, the susceptibility of these isolates to antifungal drugs was evaluated using VITEK 2 automated antifungal susceptibility testing (AFST) system. In this case report, we presented six cases of term neonates who were admitted due to respiratory distress. The neonate received numerous antibiotic courses as well as nutrients from a parenteral source due to severe enteral nutrition intolerance. Notably, the four patients did not undergo any invasive procedures; they received only mechanical ventilation support. Subsequently, one patient (Case 6) underwent surgery, and another (Case 5) remained on a ventilator. Neither of the patients who underwent invasive procedures survived. Further, all the *W. anomalous* isolated from NICU cases were susceptible to fluconazole, voriconazole, and amphotericin B.

**Conclusion:**

Our case serves as a reminder to maintain a higher level of suspicion for fungal infections in preterm and term infants in the NICU. Antifungal prophylaxis may be considered for these patients if one or more risk factors are present. Additionally, it is important to note that NAC species can occasionally be the causative agent. Therefore, we should always bear in mind the possibility of these fungal pathogens as infectious agents in neonates with risk factors.

## 1. Introduction

Neonatal sepsis is a systemic infection in newborns within the first month of life. It is a severe condition with a higher rate of mortality, thus it requires prompt recognition and treatment. [1] Fungal infections in neonates are generally less common than bacterial infections, but can still have significant morbidity and mortality rates. [2] Invasive candidiasis (IC) is a substantial source of morbidity and mortality in the neonatal intensive care unit (NICU) and the third most frequent cause of late‐onset sepsis in very‐low‐birth‐weight (VLBW) newborns. The incidence of neonatal candidiasis in low‐birth‐weight (LBW) infants ranges from 7% to 20% due to their increased vulnerability and susceptibility to infections. [3, 4] For IC, the primary etiological agent is *C. albicans*, but also other nonalbicans *Candida* (NAC) species, such as *C. parapsilosis*, *C. tropicalis*, *C. glabrata*, *C. krusei*, and *Wickerhamomyces anomalous*, which invade the bloodstream and spread to other organs in the body. [5] Neonates, particularly those in the NICU, are at increased risk for IC due to prematurity, LBW, prolonged hospitalization, and invasive medical interventions like central venous catheters or mechanical ventilation. [6–8] Maternal colonization with *Candida* during pregnancy or delivery can also contribute to the risk. [9]

In a recent scenario, the outbreak of *C. pelliculosa* fungemia in a NICU presents a significant challenge in healthcare settings. [10] *C. pelliculosa*, formerly known as *C. utilise*, *Hansenula anomala*, *Pichia anomala*, or *W. anomalous*, is a yeast commonly found in the environment, including soil, water, and food. [11] It is an opportunistic fungal pathogen that can cause invasive infections, particularly in vulnerable individuals such as neonates who are already immunocompromised due to premature birth or underlying health conditions. [12–14] The first instance of *C. pelliculosa* infection was in a baby who died from interstitial pneumonia in 1953. [15] Since then, a few isolated cases of *C. pelliculosa* infection have occurred, mostly in newborns, kids, and people with impaired immune systems. Previous studies have documented hospital‐acquired *C. pelliculosa* infections in hematology wards, surgical intensive care units, pediatric intensive care units, nurseries, and pediatric intensive care units, indicating that low gestational age, LBW, prolonged hospitalization, sustained blood alkalosis, inappropriate use of antibiotics, and invasive surgeries may increase the risk of *C. pelliculosa* infection. [11, 16, 17] This case report series presents six cases of *W. anomalous* (formerly *C. pelliculosa*) sepsis in neonates admitted to our NICU.

## 2. Case Report

### 2.1. Case 1

A full‐term male baby weighing 2.6 kg was born to a primi mother after 38 weeks of gestation. The baby was delivered via normal vaginal delivery (NVD) and did not cry immediately after birth. On the fifth day, the baby developed the first convulsion and was unable to maintain adequate oxygen saturation levels through nasal prongs and continuous positive airway pressure (CPAP) support was administered. On admission, the baby was febrile and diagnosed with clinical sepsis and severe hyperbilirubinemia. Thus, empirical antibiotic therapy with meropenem and vancomycin was initiated, followed by prophylactic fluconazole. However, the baby experienced a second convulsion, and an MRI revealed hypoxic ischemic encephalopathy. Blood cultures collected during hospitalization showed the presence of *W. anomalous*, identified by the VITEK 2 yeast identification system. The isolates were susceptible to the antifungal tested. Following antifungal therapy and supportive management, the baby was hemodynamically stable and thus discharged after 19 days (Tables 1 and 2).

**Table 1:** Clinical features of six patients with *Wickerhamomyces anomalous* (formerly *Candida pelliculosa*) isolated from blood culture.

Case	1	2	3	4	5	6
GA (weeks)/sex	38/M	38/F	38/M	35/F	38/F	38/M
BW (kg)	2.6	2.5	2.8	1.3	2.8	2.8
Diagnosis	1st convulsion and respiratory distress	Respiratory distress with subcoastal retractione	At the time birth: congenital heart disease and 4 limbs; respiratory distress	Respiratory distress	Respiratory distress	Respiratory distress and suspecting tracheoesophageal fistula
Date of admission (dd‐mm‐yy)	19‐11‐2022	05‐12‐2022	06‐12‐2022	13‐12‐2022	06‐11‐2022	16‐11‐2022
Date of discharge/death (dd‐mm‐yy)	^A^07‐12‐2022	^A^06‐12‐2022	^A^11‐12‐2022	^A^18‐12‐2022	^B^05‐12‐2022	^B^30‐11‐2022
Days of hospital stay (days)	19	2	6	5	31	15
Date of Blood culture collection (dd‐mm‐yy)	^a^29‐11‐2022	05‐12‐2022	06‐12‐2022	14‐12‐2022	^a^03‐12‐2022	^a^29‐11‐2022
Date of blood culture bottle flagging positive (dd‐mm‐yy) and cultured	04‐12‐2022	12‐12‐2022	12‐12‐2022	17‐12‐2022	12‐12‐2022	04‐12‐2022
Date of blood culture reported (dd‐mm‐yy)	06‐12‐2022	14‐12‐2022	14‐12‐2022	19‐12‐2022	14‐12‐2022	06‐12‐2022
Fungemia detected day after blood collection (days)	6	8	7	4	10	6
Fungal organism isolated^b^	*Wickerhamomyces anomalous*	*W. anomalous*	*W. anomalous*	*W. anomalous*	*W. anomalous*	*W. anomalous*
Any other organism isolated	No	No	No	No	CONS	*Pseudomonas* spp.
Mode of mechanical ventilation (CPAP/HFNC)	Yes	Yes	Yes	Yes	Yes	Yes
Invasive procedure	No	No	No	No	Ventilator	Surgery
Antibiotics treatment	Meropenem and vancomycin	Cefotaxime and amikacin	Cefotaxime and amikacin	Cefotaxime and amikacin	Cefotaxime, amikacin, and vancomycin	Cefotaxime, amikacin, aigecycline, and aiprofloxacin
Antifungal treatment	Fluconazole	No	Fluconazole	No	Amphotericin B	Fluconazole and amphotericin B
Hb (gm%)	17.5	12.8	14.9	19.1	12.8	14.7
WBC (cu.mm)	13800	31100	9400	12500	7200	12700
Platelets (Lac/cu mm)	0.56	1.89	1.50	2.57	0.20	0.21
Thrombocytopeniac	Yes	Yes	Yes	No	Yes	Yes
Febrile at the time of hospitalization	Yes	Yes	Yes	Yes	Yes	Yes
Nil by mouth	No	No	No	No	Yes	Yes
Outcome	Discharge	Discharge	Discharge	Discharge	Death	Death
Cause of death	NA	NA	NA	NA	Pulmonary hemorrhage, sepsis with DIC, and birth asphyxia	Severe persistent pulmonary hypertension, late onset of sepsis with DIC, and operated case of Type C tracheoesophageal fistula

^a^Initial blood culture shows no growth and second blood culture showed growth of organism.

^b^Identification of the organism performed by VITEK 2 yeast identification system.

^A^Date of discharge.

^B^Date of death.

**Table 2:** Antifungal susceptibility pattern of *Wickerhamomyces anomalous* (formerly *Candida pelliculosa*) performed by using VITEK 2 automated antifungal susceptibility system.

Case	Fluconazole (*μ*g/mL)	Voriconazole (*μ*g/mL)	Amphotericin B (*μ*g/mL)
1	2	0.25	<=0.25
2	2	<=0.12	<=0.25
3	2	<=0.12	<=0.25
4	2	0.25	0.5
5	2	0.25	<=0.25
6	2	0.25	0.5

### 2.2. Case 2

A full‐term female baby weighing 2.5 kg did not cry immediately but responded after receiving stimulation. Due to respiratory distress with subcostal retractions, the baby was transferred to the NICU and provided oxygen support through nasal prongs. Subsequently, the baby was admitted to our center and diagnosed with meconium aspiration syndrome. Upon admission, the blood cultures were sent for microbiological investigation and the baby was given cefoxitin and amikacin injection, along with intravenous fluids. Blood cultures collected on the same day of admission showed the presence of *W. anomalous*. The baby′s condition improved, and oxygen saturation was maintained during the transition from nasal prongs to leak oxygen. Additionally, the baby started receiving oral feeds and demonstrated good tolerance. As a result, the baby was deemed stable and subsequently discharged (Tables 1 and 2).

### 2.3. Case 3

A full‐term male baby was born through cesarean section in our rural tertiary care center. The baby was shifted to the NICU after birth and was suspected of having congenital heart disease and discrepancies in saturation levels in all four limbs. At around 4 h of age, the baby exhibited grunting and chest retractions, and an arterial blood gas analysis showed signs of acidosis. As a result, the baby was intubated and placed on ventilator support. Injections of ampicillin and gentamicin were administered due to the development of severe persistent pulmonary hypertension of the newborn (PPHN), and the baby was started on intravenous sildenafil. Despite treatment, the baby continued to experience continuous rapid breathing (tachypnea). Consequently, the baby was admitted to our center for further management. Upon admission, the baby remained intubated and showed no signs of activity. Despite treatment with ceftriaxone, amikacin, sildenafil, and prophylactic fluconazole, the infant remained critically ill. Blood culture grew *W. anomalous*, which was susceptible to amphotericin B, fluconazole, and voriconazole. Although the infant did not show significant clinical improvement, the parents decided on discharge after 6 days of hospitalization (Tables 1 and 2).

### 2.4. Case 4

A preterm female baby weighing 1.3 kg was born via cesarean section. At the time of birth, the baby exhibited respiratory distress. Upon NICU admission to another private hospital, the baby was administered oxygen via nasal prongs. At admission to our center, the baby was at a normal body temperature, was active, and showed no signs of respiratory distress or a history of grunting. The baby was closely monitored while receiving oxygen support and administered cefotaxime injections and amikacin. Blood cultures collected at admission at our center revealed growth of *W. anomalous*. After observing the baby′s condition for 5 days and ensuring vital signs and hemodynamic stability, the decision was made to discharge the baby from the hospital (Tables 1 and 2).

### 2.5. Case 5

**Figure 1:**
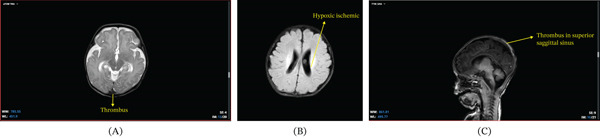
Representing the MRI report of Case 5: (A) thrombus, (B) hypoxic ischemic, and (C) thrombus in superior sagittal sinus.

A full‐term, 2.8 kg neonatal female was delivered vaginally and developed birth asphyxia, respiratory failure, and required mechanical ventilation support. While being hospitalized, the baby developed thrombocytopenia, coagulopathy, seizures, pulmonary oedema, shock, cerebral oedema, and persistent elevation of inflammatory markers. Blood culture revealed coagulase‐negative staphylococci, whereas a repeat blood culture revealed the fungus *W. anomalous* (Tables 1 and 2). The MRI scan showed evidence of hypoxic ischemic encephalopathy with superior sagittal sinus thrombosis (Figure 1). Despite the treatment with meropenem, colistin, tigecycline, ciprofloxacin, and amphotericin B, as well as repeated transfusions and supportive therapy, the patient developed pulmonary bleeding, disseminated intravascular coagulation, and septic shock. The baby passed away after prolonged resuscitation attempts.

### 2.6. Case 6

The baby was born at full term, weighing 2.8 kg, and was born via cesarean delivery. The baby had breathing problems at birth and was found to have a Type C tracheoesophageal fistula. He was referred for corrective surgery, which included right posterolateral thoracotomy. His recovery was marked by complications such as *Pseudomonas* sepsis, severe pulmonary hypertension, pulmonary haemorrhage, thrombocytopenia, and shock necessitating mechanical ventilation and blood product transfusion. The patient was on fluconazole prophylaxis, and later a repeat blood culture revealed *W. anomalous*. Treatment was further escalated by giving amphotericin B, but unfortunately, septic shock, disseminated intravascular coagulation, and death occurred after cardiopulmonary resuscitation failure (Tables 1 and 2).

## 3. Discussion

Neonatal candidiasis, a condition characterized by a fungal infection in newborns, has been observed to have mortality rates ranging from 30% to 60%. The mortality rates tend to rise as the birth weight decreases [3, 25]. Although *Candida albicans* is the most commonly implicated organism in severe fungal infections, other nonalbicans *Candida* species have also emerged as significant pathogens linked to opportunistic infections [10, 26]. Upon reviewing the literature, it was observed that outbreaks of *C. pelliculosa* fungemia primarily occur in pediatric wards [4, 27]. *C. pelliculosa* is a yeast commonly encountered in diverse sources such as fruits, tree exudates, soil, vegetables, industrial or pilot plant fermentations, and other organic materials [28]. Wang and Schwarz [29] first documented an infection involving *C. pelliculosa* in 1958 when they discovered its presence in the lungs of infants. In 1986, the first recorded epidemic occurred in a NICU, where 52 newborn infants were found to be colonized with *C. pelliculosa*. Among them, eight infants developed infections, with five experiencing fungemia, two having fungemia accompanied by ventriculitis, and one having ventriculitis alone [12] (Table 3). To date, *C. pelliculosa* has been implicated in nosocomial cerebral ventriculitis in LBW neonates, endocarditis in intravenous drug abusers, and urinary tract infections in renal transplant recipients [12, 30–32]. In the pediatric population, *C. pelliculosa* is known to cause severe nosocomial bloodstream infections, primarily as outbreaks in pediatric intensive care units [10].

**Table 3:** Review of literature of *Candida pelliculosa*.

Author	No. of patient with *C. pelliculosa*	Birth weight	Preterm/term baby	Sample	Type of study	Outbreak	Outcome	Treatment	Diagnosis
Murphy et al. 1986 [12]	8	VLBW	Preterm	Blood, CSF	Case report	No	4 survived, 2 died, 2 special care baby unit	Combination of 5‐flucytosine and amphotericin B	Asphyxia, HMD, IVH, BPD, CHD, hydrocephalus, and ventriculitis
Sekhon et al. 1992 [18]	1	NBW	Term	Blood	Case report	No	Survived	Combination of 5‐flucytosine and amphotericin B	Born with multiple congenital abnormalities, including gastric and gastrointestinal defects (hematemesis)
Wong et al. 2000 [19]	1	VLBW	Preterm	Blood	Case report	No	Not survived	Initially amphotericin B and later oral ketoconazole	Perinatal asphyxia and respiratory distress syndrome
Chakrabarti et al. 2001 [20]	379	NA	Preterm	Swab from mouth, rectum, umbilicus, and groin	Case control study	Yes	NA	NA	NA
Hanzen et al. 2002 [21]	1	NA	NA	Blood	Case report	No	NA	Oral antifungal prophylaxis	Bacteremia from a urinary source
Pasqualotto et al. 2005 [22]	17	NA	NA	Blood	Case control study	Yes	NA	All patient treated with amphotericin B except two case	NA
Paul et al. 2006 [23]	1	VLBW	Preterm	Blood	Case report	No	Not survived	Amphotericin B	Respiratory distress syndrome
Da Silva et al. 2012 [24]	5	VLBW (4 case), NBW (1 case)	Preterm	Blood	Case report	No	Survived	Fluconazole alone (3 cases); combination of fluconazole and amphotericin B (2 cases)	Congenital malformation, neonatal cholestasis, jaundice and anemia, respiratory distress syndrome, and meningoencephalitis

In this case report, we presented six cases of term neonates who were admitted due to respiratory distress. The neonates received multiple courses of antibacterial antibiotics and required parenteral nutrition due to severe enteral nutrition intolerance. Notably, the four patients did not undergo any invasive procedures only receiving mechanical ventilation support and subsequently one patient (Case 6) underwent surgery, whereas another patient (Case 5) was on ventilator. Both patients who underwent invasive procedures did not survive. In the treatment of *C. pelliculosa* fungemia, favorable antifungal activity has been observed with fluconazole, voriconazole, amphotericin B, and caspofungin [33]. Previous reports have shown effective treatment of *C. pelliculosa* fungemia with amphotericin B, either alone or in combination with 5‐flucytosine or fluconazole [13, 18, 20, 22, 34]. Similarly, our patients, with the exception of two fatal cases, were treated with amphotericin B (Case 5) and fluconazole and amphotericin B (Case 6), and their isolates exhibited susceptibility to this antifungal agent. Notably, the MIC values of the isolates do not differ between the survivors and death cases. The optimal breakpoints for amphotericin B therapy in *C. pelliculosa* fungemia have not been firmly established, but amphotericin B appears to be an effective treatment option [35]. But in our case, irrespective of usage of amphotericin B in fatal cases the patients did not survive.

A literature review conducted by Kalkancı et al. in 2010 [4] reported a total of 31 cases and epidemics involving *C. pelliculosa*. Subsequently, additional reports included two cases of meningitis in HIV patients, fungemia in sickle‐cell anemia patients, and two more NICU epidemics. The data collected indicated a total of 11 epidemics and 24 case reports, with 9 occurring in pediatric units and 2 in adult patient units. It is worth noting that although *C. pelliculosa* fungemia tends to cause more epidemics in pediatric units, it is primarily observed as sporadic cases in adults.

In NICU epidemics, Murphy et al. [12] identified colonization with *C. pelliculosa* in the skin and mucosal areas (skin, rectum, and oropharynx) of 52 neonates, accounting for 10% of the patients admitted to the ICU. Out of these colonized infants, eight developed infections. Another study reported *C. pelliculosa* colonization in 28% of 50 premature neonates, primarily in the abdominal region, mouth, and rectum, with 20% of them developing fungemia [20]. The spread of *C. pelliculosa* in nosocomial epidemics is believed to occur through cross‐contamination via the hands of healthcare personnel, starting from the first contaminated or infected patient in the hospital [4, 20]. However, proving this hypothesis is challenging unless patients are screened upon admission. Chakrabarti et al. [20] demonstrated in their study that *C. pelliculosa* strains could be transmitted from the pediatric emergency department to the neonatal ICU via the hands of healthcare personnel, as indicated by positive hand cultures.

Among the six infected infants, two died after the onset of fungemia, whereas the remaining three had normal clinical findings upon discharge and one patient discharged upon parent request. Previous reports of outbreaks involving *C. pelliculosa* have shown varying mortality rates, with some cases resulting in deaths. Breakthrough fungemia has been reported in immunocompromised patients receiving fluconazole prophylaxis, highlighting the need for alternative treatment options. Although there is limited data on voriconazole for *C. pelliculosa* infections, our study isolated *C. pelliculosa* were susceptible to voriconazole and also indicated as promising agents in the treatment of *C. pelliculosa* fungemia in neonates [8, 37].

In literature, findings suggest the prompt treatment with amphotericin B, with or without flucytosine, has been effective in improving outcomes [13, 18, 20, 22, 34]. However, in our study, the alone use of fluconazole was found to be effective but in two cases, even the use of amphotericin B could not improve the condition of the patients and patient not survive. This opportunistic pathogen primarily affects immunocompromised patients, including preterm infants, and can lead to high mortality if not promptly treated [8].

In the present case series, the routine screening for *Candida* colonization among neonates referred from outside healthcare facilities is not implemented in our NICU. However, in the future, colonization surveillance may become an essential preventive strategy in many NICUs worldwide and help identify high‐risk neonates early. In the present case series, true fungemia was identified in Cases 1 and 3, as these cases showed clinical worsening despite antibiotic therapy but improved while receiving antifungal treatment. Case 2, a symptomatic infant requiring NICU admission and oxygen support due to respiratory distress, and also having a history of antibiotic exposure and other NICU risk factors, was considered clinically significant fungemia. Case 4, although clinically stable, was a preterm infant with LBW and additional NICU‐associated risk factors.

This report emphasizes the clinical significance of emerging NAC species in the NICU. Dealing with such uncommon pathogens becomes challenging as their unfamiliarity hinders source identification and transmission control. Fungal infections in neonates can present with various symptoms, leading us to empirically treat fungemia based on clinical suspicion and risk factors. All six infants in this study exhibited nonspecific clinical signs, particularly respiratory distress and five of the infected infants showed early laboratory findings of thrombocytopenia.

The present study has certain limitations. Molecular confirmation of the isolates was not possible due to the unavailability of sequencing facilities in our laboratory. Yeast identification was performed using the automated VITEK 2 system, which is routinely used in our setting. Likewise, antifungal susceptibility testing (AFST) was performed using the VITEK 2 automated platform. In our study, all isolates were identified using the automated VITEK 2 system, which is routinely used in our laboratory for yeast identification. Similarly, AFST was also performed using the VITEK 2 automated system.

## 4. Conclusion

In summary, our cases highlight the need for a higher level of suspicion for fungal infections not only in preterm infants but also in term neonates admitted to the NICU. Antifungal prophylaxis may be considered in patients with multiple established risk factors, including antibiotic exposure, NPO status, mechanical ventilation, and surgical intervention. Importantly, NAC species may occasionally cause infection. Therefore, fungal etiologies should always be regarded as potential infectious agents in vulnerable neonates. To reduce the burden of neonatal candidiasis, strengthened infection‐prevention strategies in the NICU, including colonization screening for outside‐admission cases, may be beneficial.

## Funding

This work was supported by the Datta Meghe Institute of Higher Education and Research.

## Ethics Statement

Case series were conducted in accordance with institutional ethical standards.

## Conflicts of Interest

The authors declare no conflicts of interest.

## Data Availability

The data that support the findings of this study are available from the corresponding author upon reasonable request.
